# Dental Biofilm Removal and Bacterial Contamination of a New Doubled-Side Thermoplastic Polyurethane-Based Toothbrush: A Crossover Study in Healthy Volunteers

**DOI:** 10.3390/antibiotics11101296

**Published:** 2022-09-22

**Authors:** Ignacio Zúñiga, Margarita Iniesta, Leire Virto, Honorato Ribeiro-Vidal, Andrea Alonso-Español, Fernando Hernández, John Jairo Cardona, Anushiravan Maher-Lavandero, Bettina Alonso, Mariano Sanz, David Herrera

**Affiliations:** 1 ETEP Research Group (Etiology and Therapy of Periodontal and Peri-implant Diseases), Faculty of Dentistry, Complutense University, 28040 Madrid, Spain; 2 Department of Anatomy and Embryology, Faculty of Optics, Complutense University, 28040 Madrid, Spain; 3 Department of Periodontology, Faculty of Dentistry, University of Porto, 4200-393 Porto, Portugal

**Keywords:** dental biofilm, toothbrush, bacterial contamination, plaque index

## Abstract

Multiple toothbrush designs have been developed to enhance dental biofilm removal and decrease bacterial contamination and retention over time. Therefore, the aim of this clinical study was to compare the efficacy of a prototype of a new double-sided thermoplastic polyurethane-based toothbrush with that of a conventional nylon-bristle toothbrush. A crossover study was conducted in systemically healthy volunteers (n = 24) for two one-week periods plus one washout week. As outcome variables, plaque and gingival indices, total bacterial contamination of the toothbrushes by quantitative polymerase chain reaction (qPCR), and patient-reported outcomes were measured. Clinical and microbiological variables were analysed using a general linear model and Friedman and Wilcoxon signed-rank tests. No statistically significant differences between toothbrushes were detected neither for full-mouth PlI (*p* > 0.05) nor for GI (*p* > 0.05). Similarly, no statistically significant differences were detected for bacterial contamination after 40 seconds or 1 week of use, with results expressed either in CFU/mL or in CFU/mm^2^ (*p* > 0.05). In conclusion, the tested prototype toothbrush was as effective and safe as the control toothbrush, and the participating subjects did not experience any adverse effects from its use and rated its efficiency and effectiveness in cleaning their teeth as satisfactory.

## 1. Introduction

Over the past few decades, an increasing general awareness in the value of personal oral hygiene has become evident [1], mainly through the mounting evidence that bacterial accumulations forming biofilms on teeth and oral tissues cause the most relevant oral diseases (e.g., caries, gingivitis, periodontitis, among others). Mainly in adults, periodontitis is a major public health problem, affecting more than 50% of the population, and its severe forms represent the sixth most prevalent disease worldwide [2]. Periodontitis is not only the main cause of tooth loss in adults, but also negatively affects masticatory function and aesthetics [3], leads to disability, impairs quality of life and is a source of social inequality [4].

The prevention of these diseases (periodontitis and caries) is based on supragingival dental biofilm control [5] by either mechanical or chemical means [6], thus reducing the pathogenic microorganisms present in the biofilms [7]. Therefore, active biofilm removal at regular intervals is necessary, with mechanical toothbrushing being the most widely used method for this purpose [8]. Despite the existence of a large number of different toothbrush designs and brushing methods described in the literature, there is no consensus on the ideal toothbrush, nor on the ideal technique for either the general population or for populations with special needs [9]. The toothbrush biofilm removal ability, in terms of quality and quantity, may be influenced by several factors. Some are based on the individual, such as motivation and skills, which largely depend on an adequate oral hygiene education, including brushing methodology, duration and frequency [10]. Others, however, depend on the toothbrush type, including its size, which allows it to reach all areas of the mouth, and the material and strength of the bristles, as well as its design. Most commercially available toothbrushes are single-headed toothbrushes with nylon bristles. However, it is still unclear whether different toothbrush designs, the control of toothbrush contamination and/or materials may have a significant impact on biofilm removal. In fact, randomised controlled clinical trials evaluating eleven different manual toothbrushes did not show significant differences in biofilm removal [11].

In the search of new materials and designs, thermoplastic polyurethane (TPU) is a biocompatible and biodegradable elastomer exhibiting remarkable chemical stability and good mechanical properties [12]. It has been approved by the Food and Drug Administration (FDA) for use in different biomedical devices, such as catheters, vascular grafts and drug delivery carriers. Recently, a new TPU-based toothbrush with an advanced double-sided head and a rotating handle that reaches and adapts to the tooth anatomy has recently been designed and patented but has not been tested in clinical studies (Balene^®^, Ziz Dental Care, S.L., Madrid, Spain).

Another important element in the mechanical control of biofilm using toothbrushes is whether these devices may become contaminated by bacteria and hence become a source of infection. There is evidence that toothbrush bristles may become contaminated with microorganisms from the oral cavity, the surrounding environment or both [13,14], and this contamination increases with its use [15]. These microorganisms can remain viable even weeks after brushing [16], being favoured by the accumulation of debris and moisture on the bristles, and by the use of the hood during storage, which increases bacterial survival and retention [17]. The consequence of these microorganisms being able to survive on toothbrushes is their transmission back to the user and the cause of infections [18]. In fact, there are reports evidencing the presence of relevant amounts of opportunistic and pathogenic microorganisms, which might promote respiratory, gastrointestinal, cardiovascular and renal conditions [19]. Studies have confirmed that oral injuries may be aggravated by a contaminated toothbrush, compared to the use of sterile toothbrushes, and these injuries may even cause septicaemia after brushing [20].

Different methods for contamination control of a toothbrush have been described, such as their immersion in chlorhexidine digluconate, the use of dentifrices with antibacterial agents, the use of tetrasodium ethylenediaminetetraacetic acid (EDTA), microwaving while immersed in water and ultraviolet sanitization devices. However, these methods of toothbrush disinfection are expensive and cannot be easily implemented, so toothbrushes with inherent antibacterial properties are sought [19].

Therefore, the primary aim of this study was to evaluate and compare the clinical efficacy of a new double-sided TPU-based toothbrush with that of a conventional nylon-bristle toothbrush in terms of biofilm control, evaluated by its impact on plaque and gingival indices after a single use and after one week of use. As a secondary aim, the impact of this novel toothbrush on bacterial contamination and on subject acceptability was evaluated, compared with the use of a standard toothbrush design.

## 2. Materials and Methods

### 2.1. Study Design

**Figure 1:**
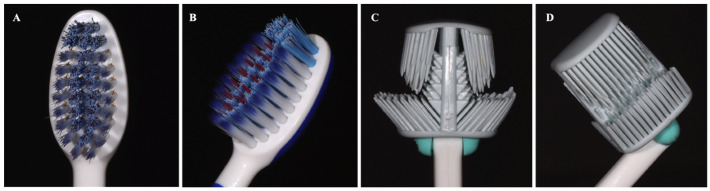
Photographs of control (**A**,**B**) and test (**C**,**D**) toothbrush heads.

A single-centre, double-blind, crossover clinical study was carried out in healthy volunteers. The test toothbrush was a double-sided TPU-based toothbrush with an advanced design (prototype of Balene^®^, Ziz Dental Care S.L., Madrid, Spain), compared to a conventional commercially available single-headed nylon-bristle toothbrush (Sensodyne Encías^®^, GSK Consumer Helthcare S.A., Madrid, Spain). See Figure 1.

### 2.2. Ethical Aspects

This study complies with the ethical principles of the Declaration of Helsinki (Edinburgh revision of October 2000), with the Standards of Good Clinical Practice and with the Spanish Code of Ethics. The research protocol was approved by the ethics committee of the CEIC Hospital Clínico San Carlos, Madrid, Spain, with the registration number 21/263-EC_X.

### 2.3. Study Population

Subjects included in the study were second-year dental students from the Faculty of Dentistry at the Complutense University of Madrid, Spain, aged between 18 and 30 years. The study was conducted from April to May 2021. Each student was invited to participate in the research after receiving written and oral detailed information from one of the investigators, about the characteristics of the study, including the potential risks and benefits of participating in the study. Subjects were enrolled if they fulfilled the following inclusion and exclusion criteria after signing the approved informed consent form:

#### 2.3.1. Inclusion Criteria

- Subjects in the range of 18–30 years old.
- Subjects who brush their teeth regularly (once or twice daily).
- Presence of at least three evaluable teeth in each quadrant.
- No interproximal attachment loss of ≥3 mm in ≥2 non-adjacent teeth [21].
- Systemically healthy.
- Non-smokers (never smokers or former smokers for at least 6 months).
- No orthodontic bands or removable prostheses.
- Subjects willing to participate and comply with the requirements of the study.

#### 2.3.2. Exclusion Criteria

- Subjects currently undergoing active dental treatment.
- Subjects currently undergoing orthodontic therapy or wearing occlusal bite guards.
- Subjects suffering from any systemic disease or condition which may affect the response of gingival tissues or the ability to perform adequate plaque control (pregnancy, diabetes, quantitative and/or qualitative polymorphonuclear neutrophils defects, other immune system disorders, etc.)
- Subjects taking medications that could interfere with the gingival tissue response (i.e., anti-inflammatory agents, diphenylhydantoin, calcium channel blockers, cyclosporine A, immunostimulants/immunomodulators).
- Subjects taking antibiotics, using antiseptics or probiotic oral health products in the previous month.

### 2.4. Randomisation and Blinding

Volunteers were allocated to one of the two treatment sequences (AB or BA, with A being the control toothbrush and B the test) with a computer-generated randomisation list using a randomised block size of six. Block randomisation and implementation of assignments were carried out by two different investigators with no clinical involvement in the trial. The order of toothbrush use and allocation were blinded to the calibrated examiners and the statistician who analysed the data.

### 2.5. Study Visits and Interventions

Once enrolled in the study, all participants were instructed to attend the baseline visit after 12 h of suspended oral hygiene and were given an appointment.

#### 2.5.1. Day 1: First Round, Baseline Visit

Two calibrated examiners (A.A.-E. and F.H.) assessed plaque (PlI) and gingival indices (GI) in all subjects. Then, they were randomly assigned to use a specific toothbrush (test or control) and were instructed in its use. Subsequently, a third examiner (I.Z.) monitored the study subjects during a single supervised toothbrushing exercise consisting of a 40 s brushing time using a sodium fluoride (equivalent to 1450 ppm fluoride ions) dentifrice (Colgate Total^®^, Colgate-Palmolive, New York, NY, USA). After this single supervised brushing exercise, PlI was re-evaluated, and the assigned brush used by the subject was retrieved and processed for microbiological contamination in the laboratory. Each subject was then given another identical toothbrush with the recommendation to use it with the same dentifrice for one week and without any other oral hygiene measures (interdental, chemical, etc.). These oral instructions were supplemented by an explanatory sheet providing all the details.

#### 2.5.2. Day 7: First Round, Final Visit

Seven days after the baseline visit, all subjects returned to the clinical research centre for the second visit of the first round, where PlI and GI were re-measured by the same examiners. Then, the subjects returned their assigned toothbrush for microbiological analysis in the laboratory and were given a questionnaire to report on their acceptance and tolerance of the toothbrush used.

#### 2.5.3. Washout Period

Due to the crossover design of this study, a one-week washout period was set up between the two study rounds. After this period, the same methodology was used for the evaluation of the other toothbrush, as each subject was his or her own control.

#### 2.5.4. Day 1: Second Round, Baseline Visit

The same process was used for the evaluation of the other toothbrush.

#### 2.5.5. Day 7: Second Round, Final Visit

The same process was used for the evaluation of the other toothbrush.

### 2.6. Clinical Outcome Variables

The main outcome variables were:

- PlI, assessed by the Turesky et al. [22] modification of the Quigley and Hein index [23], scored at six sites per tooth, after using a disclosing solution (PlacControl^®^, Dentaid, Barcelona, Spain).
- GI, assessed by the Gingival Bleeding Index [24] and by dichotomous assessment of bleeding after gentle probing, at six sites per tooth.

In addition, a comprehensive and structured visual inspection of the gingival tissues of the mouth was carried out to identify possible lesions at the gingival margin or adjacent mucosa. When the patient reported any discomfort or a change in colour and/or texture was observed, an intraoral photograph was taken for further evaluation.

### 2.7. Microbiological Analysis of Toothbrush Heads

Toothbrushes were collected after the single supervised use (day 1) and after seven days of home use (day 7) in both the first and second rounds of the study and were immediately sent to the oral microbiology laboratory for analysis.

The toothbrush heads were separated from the handle using a cold clean cutting tool (Russian 205 mm steel–vanadium pliers) to prevent material shavings during cutting and to avoid heating that could alter the composition of the material or affect the bacteria species. The toothbrush heads were transferred to tubes containing 10 mL of phosphate buffered saline (PBS) and were sonicated for 20 minutes to release the attached microorganisms. All samples were handled in a tissue-culture hood (class II biological safety), and a lysis buffer (NucliSENS Lysis Buffer, Biomerieux, France) was added to disrupt cell membranes to inactivate any possible viruses present in the sample (e.g., SARS-CoV-2).

Then, DNA was isolated using a commercial ATP Genomic DNA Mini Kit^®^ (ATP biotech, Taipei, Taiwan), following the manufacturer’s instructions, and the quantitative polymerase chain reaction (qPCR) method was used to detect and quantify total bacterial DNA.

For the amplification of total bacterial DNA present in the sample, the following methods were used:

◦ Primer 1 (forward), with sequence 5-TCCTACGGGAGGCAGCAGT-3 (350 nM).
◦ Primer 2 (reverse), with sequence 5-GGACTACCAGGGTATCTAATCCTGTT-3 (350 nM).
◦ Taqman probe, with sequence 6FAM-CGTATTACCGCGGCTGCTGGCAC-TAMRA (100 nM).

Taqman probes were labelled with the fluorochromes 6-carboxyfluorescein (FAM) at the 5’ end and 6-carboxytetramethylrhodamine (TAMRA) at the 3’ end. Quantitative qPCR amplification was performed in a total reaction mixture volume of 10 μL. The reaction mixtures contained 5 μL of 2× master mix (LC 480 Probes Master; Roche, Basel, Switzerland), optimal concentrations of primers and probe for the detection of total bacteria and 2 μL of DNA from each of the samples. Two microlitres of sterile water (Water PCR grade, Roche) was used as a negative control.

Samples were subjected to an initial amplification cycle of 95 °C for 10 min, followed by 45 cycles at 95 °C for 15 s and 60 °C for 1 min. Analyses were performed on a Light Cycler^®^ 480 II thermal cycler (Roche). The plates used in the study were FramStar 480 (4titude; The North Barn; Damphurst Lane, UK), sealed with qPCR Adhesive Clear Seals (4titude). Each DNA sample was analysed in duplicate. The quantification cycle (Cq) value was determined using the software package provided (LC480 Software1.5; Roche). Quantification of cells by qPCR is based on standard curves obtained by serial dilutions from 10^1^ to 10^9^ genomic DNA. Correlation between Cq values and colony forming units (CFU)/mL was automatically generated by the software (LC480 Software1.5; Roche).

After processing, the samples were stored at −20 °C until publication of the project results in a research journal. Subsequently, the samples will be destroyed by autoclaving and then treated as municipal waste. Microbiological outcome variables (bacterial counts in CFU) were expressed as absolute counts and as a function of head surface for each type of toothbrush.

### 2.8. Patient-Reported Outcome Measures (PROMs)

A predefined questionnaire, adapted from a previous publication [25] which included questions on subject perception, usability and adverse effects, was given to each subject, who answered it after each study period.

### 2.9. Data Analysis

#### 2.9.1. Sample Size Calculation

The sample size calculation was based on the data from the Claydon and Addy study [26] using the changes in PlI as the primary outcome, with a minimum expected effect size of 8.2% and a common standard deviation of 10%, resulting in a requirement of 24 patients to achieve an alpha risk of 0.05 and a beta risk of 0.2 in a two-sided test.

#### 2.9.2. Calibration

The primary outcome (PlI) was evaluated by two calibrated examiners (A.A.-E. and F.H.). Before the study, an inter- and intra-examiner calibration exercise was performed by recording duplicate PlI measurements in two patients, twice during the same visit at 30 min intervals, and then calculating the inter-rater reliability. Inter-examiner calibration using Cohen’s Kappa scores resulted in a percentage of agreement of 87.8%, while intra-examiner calibration was 92.6% and 93.4% for F.H. and A.A.-E., respectively.

#### 2.9.3. Statistical Analysis

For continuous data, the Shapiro–Wilk test and the distribution of data were used to assess normality. Data were expressed as means and standard deviation (SD), and as median and interquartile ranges (IQR) for nonparametric data. Categorical data were expressed as percentages.

To analyse the effect of the crossover design on the primary outcome (PlI), in spite of subject allocation by block randomisation, the difference in the change in PlI for subjects with the AB sequence and then for the BA sequence was calculated, using the Mann–Whitney U test. Subsequently, the period effect was evaluated with the Wilcoxon signed-rank test. The confidence intervals were calculated by the Hodges–Lehmann estimator.

PlI and GI were compared by repeated measures using the Friedman test to maintain the paired nature of the design, and pairwise comparisons (test versus control, baseline versus 40second data and baseline versus 1-week data) were performed with a post hoc test applying the Bonferroni correction. For microbiological data, the logarithmic transformation of CFU of bacterial counts was used to normalise the data distribution, and repeated measurements with a general linear model (GLM) were employed. The Wilcoxon signed-rank test was used to compare the participant satisfaction scores for each of the toothbrushes tested.

All statistical analyses were performed using the SPSS 25 software package (SPSS Inc., Chicago, IL, USA), and the level of significance was set at 0.05.

## 3. Results

### 3.1. Study Population

**Figure 2:**
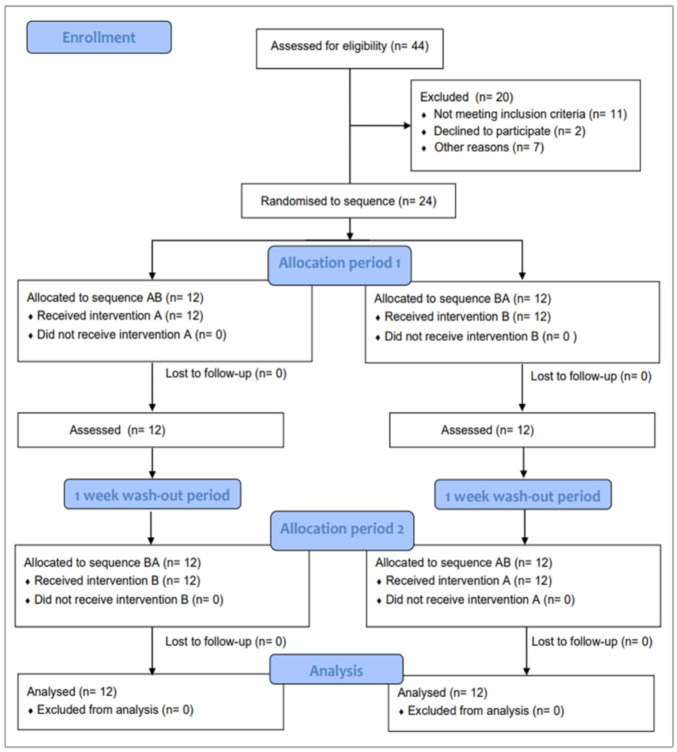
CONSORT flow chart for the crossover trial. A: control toothbrush; B: test toothbrush.

Out of 44 screened subjects, twenty-four (mean age 21.58; SD = 2.68) were randomised to a treatment sequence (12 to AB and 12 to BA sequence). All randomised subjects completed the study as depicted in the flow chart of the study (Figure 2). Table 1 shows the characteristics of the sample at baseline for the entire group and for each of the sequences.

### 3.2. Clinical Outcome Variables

There was no carry-over or period effect for the primary outcome, PlI (*p* > 0.05), (Table 2).

At baseline, there were no statistically significant differences between the test and control toothbrushes for the full-mouth PlI (*p* > 0.05), at 40 seconds (*p* > 0.05) or at 1 week (*p* > 0.05). Reductions in full-mouth PlI were observed between baseline and after 40 seconds of supervised toothbrushing for both test and control. Reductions with the control toothbrush were statistically significant for all sites (*p* = 0.009) and for buccal (*p* = 0.045) and nonproximal (*p* = 0.023) sites. Neither test nor control toothbrushes showed a significant impact on lingual and proximal sites (Table 3).

No differences in GI were observed between toothbrushes either at baseline or after one week of unsupervised use. Reductions from baseline to one week were observed in the test and control groups, although no statistically significant differences were observed (Table 4). No lesions were observed in the gingival tissue or the adjacent mucosa after one week of toothbrush use.

### 3.3. Microbiological Outcome Variables

No statistically significant differences between toothbrushes were detected after 40 seconds or 1 week of use, neither in CFU/mL nor in CFU/mm^2^ (*p* > 0.05) (Table 5).

### 3.4. Patient-Reported Outcome Measures

Table 6 depicts the mean scores of the items evaluated for each of the devices. Statistically significant differences were found for all items, with the control toothbrush receiving the best ratings. The overall mean rating on a scale from 0 to 10 for each toothbrush was 5.83 (SD = 1.94) for the test and 7.79 (SD = 2.39) for the control toothbrush, with the differences being statistically significant (*p* = 0.003).

At the end of the study, the subjects rated satisfactorily the efficiency and effectiveness of the test toothbrush in cleaning their teeth. Conversely, the interdental cleanliness and the softness of the test toothbrush were rated below five (Table 7).

## 4. Discussion

The results of the present study showed similar efficacy between the new double-sided thermoplastic polyurethane-based toothbrush and the conventional single-headed nylon-bristle toothbrush for both plaque and gingival inflammation reduction, and these results were for both the single supervised use (PlI) and one-week unsupervised use (PlI and GI). Similarly, there were no significant differences between the tested toothbrushes regarding bacterial contamination, both after a single use and after one week of use. The participating subjects did not experience any adverse effects when using any of the toothbrushes and rated satisfactorily the efficiency and effectiveness of the test toothbrush.

Due to its novelty, it is difficult to establish direct comparisons between this TPU-based toothbrush and other toothbrushes. The toothbrush design most similar to the one tested in this RCT is the triple-headed toothbrush (Superbrush^®^, Dentaco AS, Minde/Bergen, Norway), which has been tested in specific populations. Kiche et al. [27] conducted a crossover study in children, with two weeks of toothbrush use, and only found statistically significant differences in plaque levels, favouring the control toothbrush in buccal sites, although the children expressed their preference for the test toothbrush. A parallel study conducted in a mentally handicapped population found no statistically significant differences at either of the evaluation periods (one and three weeks) in terms of PlI reduction with either of the toothbrushes evaluated (Superbrush^®^ or a traditional toothbrush), although the authors highlight the ease of instruction in the use of the modified toothbrush and suggest that it can be a useful device in individuals with cognitive or physical impairment or in those for whom oral hygiene must be performed by a third person [28]. This triple-headed toothbrush (Superbrush^®^) has also been compared to two other toothbrushes (manual and powered toothbrush) also using a crossover RCT design, with one-week test periods separated by a one-week washout period. The results reported better performance of the test toothbrush, similarly in the three subject groups studied (children, dental students and adults aged 37–60 years old) [29]. A systematic review evaluating the efficacy of triple-headed toothbrushes compared with that of conventional single-headed manual toothbrushes reported no differences for self-performed brushing, while when the caregiver performed the toothbrushing, three out of four studies reported differences favouring triple-headed manual toothbrushes [30]. Another systematic review, focusing specifically on children and adolescents with intellectual disabilities, suggests that conventional toothbrushes were less effective than modified toothbrushes, although the meta-analyses showed conflicting results [31].

In this study, since the double-sided test toothbrush had not been tested before, healthy dental students who volunteered to participate were used, as this sample population has been tested before in similar studies [29], and one of the objectives was to test the safety and acceptability of the proposed prototype. This may be considered as a limitation [32], as dental students usually have higher standards and knowledge of oral hygiene procedures; however, students were selected at a very early stage of their academic career with minimal exposure to oral hygiene procedures and hence similar to a conventional young adult population.

Testing the efficacy of toothbrushes in plaque removal is complex in a short-term clinical study, as many different factors may influence the results, such as toothbrushing duration, motivation, toothbrushing frequency, manual dexterity, or the novelty effect [33,34]. To minimise the effect of these factors, the present study was designed as a crossover study, with one week of use plus a one-week washout period, also including a supervised session at the beginning of the trial. Although both the single supervised toothbrushing session and the one-week unsupervised period have been shown to assess plaque removal effectiveness, they fulfil different objectives [35]. The single supervised session helps limit the confounding variables such as cooperation, toothbrushing frequency and even the Hawthorne effect, but this design is unable to assess the changes in gingival health [26,36]. Hence, the impact of the tested toothbrush on gingival health was evaluated after one week of use, as well as by collecting further information on the changes in the PlI over time.

In the supervised brushing exercise, an arbitrary brushing time of 40 s was set, assuming that if there were differences in efficacy between the toothbrushes tested, these would be more likely within the first minute, although the results showed very similar outcomes. In addition, in the week of unsupervised brushing, the brushing time could have varied among subjects, as they were asked not to change their usual practices, except for the use of the assigned toothbrush. Classical studies have shown that when probands are left to decide, the brushing time can range between 30 s and 8 min [37]. McCracken et al. [38] studied the effect of brushing time and brushing force on plaque removal and concluded that plaque removal efficacy increased with longer brushing times. In fact, a nonstandardised brushing time of one week might have influenced the results of the present investigation. However, the brushing time of each subject was not modified to allow for higher external validity. Although one week of use may be perceived as insufficient evaluation time, the nature of the design as a crossover study allowed us to test and control the toothbrushes over each participant, and the results did not demonstrate any carry-over and period effect. In the scientific literature, there are studies that demonstrate significant efficiency over short periods (e.g., one week) that disappear after a few weeks of use [38]; other studies that report differences after one week in favour of the control toothbrush, but observe the opposite effect after four weeks [32]; or even studies in which the impact was only observed after three months of follow-up [39]. All these studies suggest that professional brushing instructions are a key contributing factor to a study’s efficacy [40]. Taking this evidence into account, in the present study, standardised instructions on the method of brushing with the test toothbrush were provided.

Bacterial contamination of toothbrushes has been associated with repeated use of a toothbrush [20] and with the design and quality of toothbrush materials and bristles [41], mainly if there is an interface between the filaments and their insertion site in the toothbrush head [42]. As the test toothbrush was designed solely and exclusively with TPU and the bristles were part of a single structure, it was hypothesised that bacterial contamination would be reduced when compared with the control toothbrush, which has a nylon bristle insertion area in the brush head that creates a favourable space for microfiltration and bacterial proliferation. However, the results from this investigation did not reveal significant differences in the bacterial load after brushing, neither after one single use nor after one week of use. On the other hand, the adhesion of microorganisms to toothbrushes also depends on the number of filaments or bristles included in each tuft or even on the number of tufts [43], in other words, on the potential surface area to be colonised. It should be noted that the design of the test toothbrush may have influenced the results as, despite having more surface area than the control toothbrush, it showed less bacterial contamination (CFU/mm^2^) than the control toothbrush in single supervised use, although without reaching statistically significance.

As explained above, the test toothbrush, with an advanced design for closed-mouth use and with right-to-left movements that simultaneously brush the buccal and lingual/palatal aspects of both arches, may offer advantages for both self-brushing and brushing by a third person, which could help children, elderly people and special-needs populations. Indeed, these are the populations most vulnerable to bacterial contamination of the toothbrushes. We could not test this hypothesis in this research, as we used a healthy young adult population with conventional oral hygiene standards.

The potential advantages of the new toothbrush design may also allow for less time to achieve an efficient mechanical control of biofilm, as the American Dental Association (ADA) [44] recommends a brushing time of at least 2 min with a manual toothbrush. In the present study, most volunteers rated the efficiency and effectiveness of the test toothbrush in cleaning their teeth as satisfactory. However, they also acknowledged that with the allocated time (40 s) for the single supervised use with the conventional toothbrush, they were only able to brush half of their dentition and never the full mouth. Conversely, with the test toothbrush, they were able to brush both arches within the stipulated time.

This clinical trial presents evident limitations as the study design is a model and not a real clinical trial, and the aforementioned factors (use of a healthy young population, arbitrarily set brushing duration, use of toothpaste and short duration of follow-up) may have prevented a more adequate evaluation of the differential efficacy of the tested toothbrush prototype. Furthermore, interpretation and comparison of the results is difficult, due to lack of direct comparisons with previous research. In addition, the test toothbrush was a prototype and not the final version which is now commercially available; additional improvements have been made in the design, materials and manufacturing process which may lead to a better performance, especially in terms of contamination.

The microbiological study also has limitations, as only the total bacterial load was evaluated, without making any attempt to characterise the microbiota. In addition, toothbrushes may present contamination even before use [20], so previous decontamination/sterilisation may be recommended [41]. Finally, the storage location of the toothbrushes during their weekly use may have affected the results by possible cross-contamination [45].

Therefore, the results obtained should be interpreted with caution. Longer studies with larger sample sizes, conducted in a population with less motivation and manual dexterity or in populations with special needs might have presented a more evident benefit of the test toothbrush.

## 5. Conclusions

Within the limitations of the present study, it can be concluded that the prototype of the tested toothbrush was as effective and safe as the manual control toothbrush. Moreover, the subjects rated the efficiency and effectiveness of the test toothbrush in cleaning their teeth satisfactorily with no adverse effects.

## Figures and Tables

**Table 1:** Demographic variables, tobacco and systemic conditions for subjects in each sequence of use, and for the whole sample.

		AB Sequence	BA Sequence	All
n	Total	12	12	24
Age	Mean (SD)	21.75 (2.89)	21.75 (2.89)	21.58 (2.68)
	Maximum	30	28	30
	Minimum	20	20	20
Gender n (%)	Male	3 (25%)	2 (16.7%)	5 (20.8%)
	Female	9 (75%)	10 (83.3%)	19 (79.2%)
Smoking n (%)	No	12 (100%)	12 (100%)	24 (100%)
	Yes	0	0	0
Systemic conditions n (%)	No	11 (91.7%)	11 (91.7%)	11 (91.7%)
	Yes	1 (8.3%)	1 (8.3%)	1 (8.3%)
Allergies n (%)	No	10 (83.3%)	11 (91.7%)	21 (87.5%)
	Yes	2 (16.7%)	1 (8.3%)	3 (12.5%)

AB: first control (A), then test (B); BA: first test (B), then control (A). n: sample size; SD: standard deviation; n (%): number of patients and percentage.

**Table 2:** Calculations for testing carry-over and period effect of the crossover trial based on the primary outcome, plaque index.

Carry-Over Effect		n	Mean	SD	Median	IQR	Median Difference	95% CI		*p*-Value
								Lower Bound	Upper Bound	
All	AB	12	−0.01	0.07	0.00	0.03	0.03	−0.01	0.13	0.219
	BA	12	−0.08	0.11	−0.02	0.17				
Buccal	AB	12	−0.04	0.09	−0.00	0.07	0.02	−0.02	0.17	0.319
	BA	12	−0.13	0.18	−0.02	0.23				
Lingual	AB	12	0.00	0.09	0.00	0.02	0.00	−0.02	0.11	0.551
	BA	12	−0.03	0.11	0.00	0.12				
Proximal	AB	12	−0.01	0.05	0.00	0.01	0.01	0.00	0.08	0.198
	BA	12	−0.07	0.10	−0.01	0.17				
Non-proximal	AB	12	−0.01	0.12	0.00	0.05	0.05	−0.01	0.21	0.198
	BA	12	−0.09	0.13	−0.04	0.24				
**Period Effect**		**n**	**Period 1**		**Period 2**		**Median Difference**	**95% CI**		***p-*** **value**
								**Lower Bound**	**Upper bound**	
All	Mean (SD)	24	0.95	0.07	0.92	9.12				
	Median (IQR)	24	0.99	0.03	0.98	0.13	−0.01	−0.06	0.00	0.185
Buccal	Mean (SD)	24	0.95	0.08	0.90	0.17				
	Median (IQR)	24	0.98	0.07	0.98	0.09	−0.01	−0.08	0.01	0.326
Lingual	Mean (SD)	24	0.96	0.08	0.94	0.09				
	Median (IQR)	24	1.00	0.02	0.98	0.10	0.00	−0.04	0.01	0.344
Proximal	Mean (SD)	24	0.97	0.05	0.94	0.10				
	Median (IQR)	24	1.00	0.02	0.99	0.08	−0.00	−0.04	0.00	0.182
Non-proximal	Mean (SD)	24	0.92	0.12	0.88	0.15				
	Median (IQR)	24	0.98	0.05	0.97	0.22	−0.01	−0.10	0.00	0.184

AB: first control (A), then test (B); BA: first test (B), then control (A). n: sample size; SD: standard deviation; IQR: interquartile range; CI: confidence interval.

**Table 3:** Plaque index analysis with the repeated measures Friedman test.

Sites		n	Test				Control				*p*-Value
			Mean	SD	Median	IQR	Mean	SD	Median	IQR	
All	BL	24	0.87	0.16	0.95	0.21	0.89	0.17	0.97	0.09	1.000
	40s	24	0.84	0.17	0.92	0.25	0.82	0.22	0.91	0.20	1.000
	1w	24	0.96	0.05	0.99	0.03	0.84	0.17	0.92	0.25	1.000
	BL	24	0.87	0.16	0.95	0.21					1.000 *
	40s	24	0.84	0.17	0.92	0.25					
	1w	24	0.96	0.05	0.99	0.03					0.418 ^†^
	BL	24					0.89	0.17	0.97	0.09	**0.009** *
	40s	24					0.82	0.22	0.91	0.20	
	1w	24					0.84	0.17	0.92	0.25	1.000 ^†^
Buccal	BL	24	0.85	0.18	0.94	0.23	0.86	0.21	0.97	0.18	1.000
	40s	24	0.78	0.22	0.86	0.36	0.77	0.24	0.88	0.33	1.000
	1w	24	0.97	0.04	1.00	0.03	0.88	0.18	0.97	0.18	1.000
	BL	24	0.85	0.18	0.94	0.23					0.559 *
	40s	24	0.78	0.22	0.86	0.36					
	1w	24	0.97	0.04	1.00	0.03					0.082 ^†^
	BL	24					0.86	0.21	0.97	0.18	**0.045** *
	40s	24					0.77	0.24	0.88	0.33	
	1w	24					0.88	0.18	0.97	0.18	1.000 ^†^
Lingual	BL	24	0.90	0.14	0.99	0.17	0.93	0.15	1.00	0.03	1.000
	40s	24	0.90	0.15	0.99	0.20	0.86	0.20	0.96	0.21	1.000
	1w	24	0.95	0.08	1.00	0.04	0.94	0.09	1.00	0.10	1.000
	BL	24	0.90	0.14	0.99	0.17					1.000 *
	40s	24	0.90	0.15	0.99	0.20					
	1w	24	0.95	0.08	1.00	0.04					1.000 ^†^
	BL	24					0.93	0.15	1.00	0.03	0.131 *
	40s	24					0.86	0.20	0.96	0.21	
	1w	24					0.94	0.09	1.00	0.10	1.000 ^†^
Proximal	BL	24	0.90	0.13	0.99	0.19	0.92	0.15	1.00	0.05	1.000
	40s	24	0.88	0.16	0.96	0.21	0.85	0.20	0.93	0.14	1.000
	1w	24	0.98	0.03	1.00	0.01	0.93	0.11	1.00	0.08	1.000
	BL	24	0.90	0.13	0.99	0.19					1.000 *
	40s	24	0.88	0.16	0.96	0.21					
	1w	24	0.98	0.03	1.00	0.01					1.000 ^†^
	BL	24					0.92	0.15	1.00	0.05	0.131 *
	40s	24					0.85	0.20	0.93	0.14	
	1w	24					0.93	0.11	1.00	0.08	1.000 ^†^
Non-proximal	BL	24	0.83	0.21	0.91	0.25	0.84	0.23	0.95	0.20	1.000
	40s	24	0.77	0.22	0.84	0.36	0.75	0.25	0.86	0.35	1.000
	1w	24	0.93	0.10	0.98	0.09	0.87	0.17	0.96	0.24	1.000
	BL	24	0.83	0.21	0.91	0.25					1.000 *
	40s	24	0.77	0.22	0.84	0.36					
	1w	24	0.93	0.10	0.98	0.09					0.508 ^†^
	BL	24					0.84	0.23	0.95	0.20	**0.023** *
	40s	24					0.75	0.25	0.86	0.35	
	1w	24					0.87	0.17	0.96	0.24	1.000 ^†^

n: sample size; SD: standard deviation; IQR: interquartile range; BL: baseline; 40s: 40 seconds; 1w: 1 week. * baseline versus 40 seconds; ^†^ baseline versus 1 week. Statistically significant differences are in bold.

**Table 4:** Gingival index analysis with the repeated measures Friedman test.

Sites		n	Test				Control				*p*-Value *
			Mean	SD	Median	IQR	Mean	SD	Median	IQR	
All	BL	24	0.13	0.19	0.03	0.14	0.11	0.15	0.02	0.19	0.063
	1w	24	0.06	0.09	0.02	0.07	0.07	0.11	0.02	0.10	
	BL	24	0.13	0.19	0.03	0.14					
	1w	24	0.06	0.09	0.02	0.07					
	BL	24					0.11	0.15	0.02	0.19	
	1w	24					0.07	0.11	0.02	0.10	
Buccal	BL	24	0.12	0.19	0.03	0.20	0.10	0.14	0.01	0.16	0.304
	1w	24	0.05	0.10	0.01	0.05	0.07	0.13	0.01	0.10	
	BL	24	0.12	0.19	0.03	0.20					
	1w	24	0.05	0.10	0.01	0.05					
	BL	24					0.10	0.14	0.01	0.16	
	1w	24					0.07	0.13	0.01	0.10	
Lingual	BL	24	0.14	0.19	0.05	0.12	0.12	0.16	0.04	0.21	0.073
	1w	24	0.07	0.09	0.02	0.09	0.08	0.12	0.02	0.09	
	BL	24	0.14	0.19	0.05	0.12					
	1w	24	0.07	0.09	0.02	0.09					
	BL						0.12	0.16	0.04	0.21	
	1w	24					0.08	0.12	0.02	0.09	
Proximal	BL	24	0.16	0.24	0.04	0.23	0.14	0.19	0.02	0.22	0.182
	1w	24	0.07	0.12	0.02	0.06	0.09	0.14	0.02	0.14	
	BL	24	0.16	0.24	0.04	0.23					
	1w	24	0.07	0.12	0.02	0.06					
	BL	24					0.14	0.19	0.02	0.22	
	1w	24					0.09	0.14	0.02	0.14	
Non-proximal	BL	24	0.07	0.11	0.02	0.07	0.06	0.09	0.01	0.09	0.228
	1w	24	0.03	0.04	0.02	0.05	0.04	0.07	0.01	0.07	
	BL	24	0.07	0.11	0.02	0.07					
	1w	24	0.03	0.04	0.02	0.05					
	BL	24					0.06	0.09	0.01	0.09	
	1w	24					0.04	0.07	0.01	0.07	

n: sample size; SD: standard deviation; IQR: interquartile range; BL: baseline; 1w: 1 week. * No multiple comparisons were made, as the null hypothesis of no differences was retained.

**Table 5:** Log 10 of colony forming units (CFU) per millilitre and per square millimetre of the toothbrush surface.

		n	Test		Control		Mean Difference	95% CI		*p*-Value
			Mean	SD	Mean	SD		Lower Bound	Upper Bound	
CFU/mL	40s	24	7.27	0.40	7.24	0.48	0.03	−0.36	0.43	1.000
	1w	24	7.42	0.32	7.10	0.68	0.32	−0.03	0.67	0.099
	40s	24	7.27	0.40			−0.15	−0.47	0.17	1.000
	1w	24	7.42	0.32						
	40s	24			7.24	0.48	0.13	−0.36	0.63	1.000
	1w	24			7.10	0.68				
CFU/mm^2^	40s	24	3.41	0.40	3.42	0.48	−0.01	−0.41	0.37	1.000
	1w	24	3.56	0.32	3.29	0.68	0.26	−0.09	0.62	0.246
	40s	24	3.41	0.40			−0.15	−0.47	0.17	1.000
	1w	24	3.56	0.32						
	40s	24			3.42	0.48	0.13	−0.36	0.63	1.000
	1w	24			3.29	0.68				

n: sample size; SD: standard deviation; CI: confidence interval; CFU/mL: colony forming units per millilitre; CFU/mm^2^: colony forming units per square millimetre; 40s: 40 seconds; 1w: 1 week.

**Table 6:** Mean, standard deviations (SD), median and interquartile range (IQR) for the scores of the items assessed for each of the toothbrushes studied (patient-reported outcome measures). The answers were provided on a 1–10 scale.

Item	n	Test				Control				*p*-Value
		Mean	SD	Median	IQR	Mean	SD	Median	IQR	
Easy to pick it up	24	8.04	1.23	8.00	2	9.29	0.99	10.00	1	**0.001**
Aesthetic appearance	24	7.08	2.28	7.50	3	8.54	1.44	8.50	2	**0.016**
Lightweight	24	8.04	1.60	8.00	2	9.04	1.36	9.00	1	**0.018**
Easy to use	24	7.54	2.16	8.00	4	8.96	1.70	10.00	1	**0.022**
Comfortable mouth use	24	5.58	2.66	5.50	5	8.88	1.77	10.00	2	**<0.001**
Access to difficult areas	24	4.13	2.45	4.50	4	7.58	2.44	8.00	3	**<0.001**
Satisfying clean feeling	24	4.63	2.22	4.50	3	7.21	3.02	8.50	4	**0.002**
Gentle on teeth	24	5.83	2.16	6.00	3	9.25	0.98	10.00	1	**<0.001**
Adjusting to irritated gums	24	5.00	2.46	5.00	4	8.54	1.38	9.00	2	**<0.001**
Overall rating	24	5.83	1.94	6.00	2	7.79	2.39	9.00	2	**0.003**

n: sample size; SD: standard deviation; IQR: interquartile range. Statistically significant differences are in bold.

**Table 7:** Mean, standard deviations (SD) and minimum (Min) and maximum (Max) scores of the evaluation of the test toothbrush at the end of the study (patient-reported outcome measures). The answers were provided on a 1–10 scale.

Item	n	Test			
		Mean	SD	Min	Max
More efficient than conventional toothbrush	24	5.63	2.49	1	10
Makes cleaning easier than conventional toothbrush	24	6.25	2.70	1	10
Cleans better between and around teeth than conventional toothbrush	24	4.96	2.25	1	9
Softer than conventional toothbrush	24	4.38	2.35	1	9

n: sample size; SD: standard deviation.

## Data Availability

All data included in this study are available upon request through contact with the corresponding author.
